# Infantile Primary Hyperoxaluria Type 1 Treated With Lumasiran in Twin Males

**DOI:** 10.7759/cureus.21673

**Published:** 2022-01-27

**Authors:** Khaled Aldabek, Oulimata K Grossman, Osama AL-Omar, Janelle A Fox, Michael L Moritz

**Affiliations:** 1 Urology/Pediatric Urology, WVU Medicine Children's Hospital/West Virginia University School of Medicine, Morgantown, USA; 2 Pediatrics/Pediatric Nephrology, WVU Medicine Children's Hospital/West Virginia University School of Medicine, Morgantown, USA; 3 Urology/Pediatric Urology, UPMC Children’s Hospital of Pittsburgh/University of Pittsburgh School of Medicine, Pittsburgh, USA; 4 Pediatrics, University of Pittsburgh School of Medicine, Pittsburgh, USA; 5 Pediatric Nephrology, UPMC Children's Hospital of Pittsburgh, Pittsburgh, USA

**Keywords:** children, twin boys, renal stone disease, lumasiran, primary hyperoxaliuria

## Abstract

Primary hyperoxaluria type 1 (PH1) is a rare genetic disease that results in oxalate overproduction leading to nephrolithiasis (NL), nephrocalcinosis (NC), kidney failure, and systemic oxalosis. Infantile PH1 is its most severe form, and it may require intensive hemodialysis followed by a liver-kidney transplant. Lumasiran is an RNA interference (RNAi) therapeutic agent that reduces hepatic oxalate production, which has been recently approved for the treatment of PH1. In this report, we present a case of twin males with infantile PH1 and bilateral NL and NC who were treated with lumasiran at 12 months of age. Their symptoms abated after therapy was started without disease progression. To the best of our knowledge, this is the first report of PH1 occurring in twins and the first report on using lumasiran to treat infantile PH1 outside of a clinical trial. Lumasiran appears to be a successful therapeutic option for infantile PH1.

## Introduction

Primary hyperoxaluria type 1 (PH1) is part of a group of rare (one to three per million population affected) autosomal recessive inborn disorders of glyoxylate metabolism caused by a deficient liver-specific enzyme, alanine-glyoxylate aminotransferase (AGT), which catalyzes the conversion of glyoxylate to glycine [1-3]. This deficiency leads to the accumulation of glyoxylate, which is metabolized to oxalate by the enzyme lactate dehydrogenase. The increase in oxalate production results in insoluble calcium oxalate crystal formation, causing nephrolithiasis (NL), nephrocalcinosis (NC), and subsequent kidney failure. The accumulation of calcium oxalate in tissues leads to systemic oxalosis [1].

PH1 can occur at any age with manifestations ranging from occasional NL to end-stage kidney disease (ESKD) and systemic oxalosis. Approximately 10% of the cases occur in early infancy and are characterized by early NC, failure to thrive, and ESKD [1]. Treatment of PH1 has been limited to hyperhydration and crystallization inhibitors, pyridoxine to reduce oxalate biosynthesis, surgical management of kidney stones, intensive dialysis to remove oxalate, and liver transplantation to correct hepatic overproduction of oxalate.

**Figure 1:**
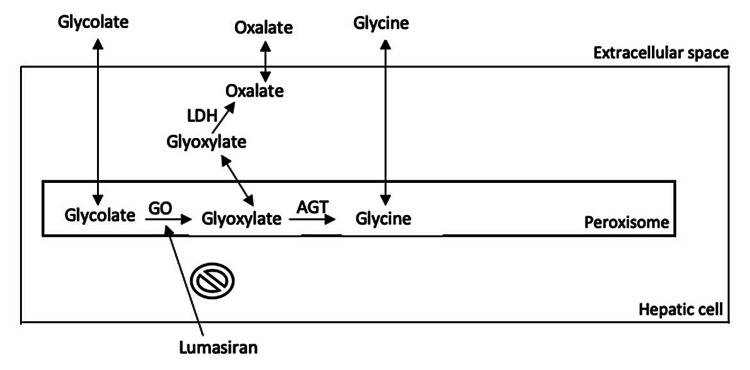
Biochemical pathway of oxalate production in liver cells and lumasiran's mechanism of action AGT: alanine-glyoxylate aminotransferase; LDH: lactate dehydrogenase; GO: glycolate oxidase

Lumasiran is a subcutaneously administered RNA interference (RNAi) therapeutic agent targeting the molecular pathway of glyoxylate metabolism, specifically directed at hepatic cells; it received approval for the treatment of PH1 in November 2020 in the USA and Europe. Lumasiran reduces glycolate oxidase (GO) by degrading the mRNA-encoding GO (Figure 1). The reduction in GO results in a decline in liver-produced oxalates [4,5]. A double-blinded randomized trial in PH1 patients aged six years or older has demonstrated a 53.5% reduction in 24-hour urinary oxalate excretion and a substantial decrease in plasma oxalate levels [4]. An open-label trial of patients <6 years with PH1 has demonstrated a 72% reduction in spot urine oxalate-creatinine ratio [6].

We discuss a case of twin males with infantile PH1 and severe kidney stone disease who were successfully treated with lumasiran. To our knowledge, this is the first report on twins with PH1 and our patients are the youngest children to be treated with lumasiran so far.

## Case presentation

This report involves dizygotic twin gestation males who were delivered via emergency C-section at 31 weeks of gestation due to preeclampsia. Twin A weighed 1.5 kg at birth and Twin B weighed 1.3 kg. They were hospitalized for approximately five weeks to optimize their feeding and promote their growth and development. Their family history was notable both for kidney stones in their father at the age of 12 years and for recurrent NL in a paternal great uncle, which had required surgical intervention.

Twin A began passing yellow-appearing crystals in his diaper following discharge from the hospital. He was brought to the emergency department at nine months of age with a kidney stone that obstructed his urethra. Ultrasound imaging demonstrated numerous bilateral renal stones, the largest measuring up to 1.0 cm, mild to moderate left hydronephrosis, and echogenic debris within the bladder. Bilateral double-J ureteral stents were placed. Six weeks later, a repeat cystourethroscopy revealed that the bilateral ureteral stents were heavily encrusted and calcified, requiring stent exchange complicated by an Enterococcus urinary tract infection.

A metabolic evaluation was within normal limits with slightly elevated serum creatinine at 0.47 mg/dl. Spot urine oxalate-creatinine ratio was elevated at 0.19 mg/mg (reference level for age one to two years: <0.103) [7]. Stone analysis revealed 100% calcium oxalate crystals (80% monohydrate and 20% dihydrate). A CT scan demonstrated multiple bilateral renal calculi, the largest measuring 7 mm, medullary NC, and ureteric calculi around the double-J stent. He underwent bilateral ureteroscopic stone extraction with laser lithotripsy and a complicated bilateral ureteral stent removal. Hydrochlorothiazide was started and then discontinued after genetic analysis confirmed PH1 with two pathogenic variants of the AGXT gene at c481G>T (p. Gly161Cys) and C.508>A (p.Gly170Arg). Potassium citrate-citric acid solution (Polycitra K) and pyridoxine were then started at 11 months of age.

Lumasiran was started at 12 months of age at a weight of 7.7 kg as subcutaneous injections at a dose of 6 mg/kg monthly for the first three months, and 3 mg/kg monthly thereafter. Polycitra K and pyridoxine were discontinued.

Before starting lumasiran, Twin A had a history of urticarial rashes associated with sun exposure, hot or cold baths, sitting on the grass, drinking, or eating. He was stoic in nature and did not display emotions. Following the start of lumasiran, it was reported that his symptoms improved, and he became more active and emotionally expressive. A follow-up serum creatinine level was found to be 0.18 mg/dl.

Twin B was screened with a renal and bladder ultrasound at 10 months of age, which demonstrated multiple small renal bilateral calculi measuring 3-4 mm without hydronephrosis. The metabolic evaluation was within normal limits except for an elevated random oxalate-creatinine ratio at 0.125 mg/mg (reference level for age one to two years: <0.103) and serum creatinine of 0.31 mg/dl. A CT scan demonstrated bilateral nonobstructive renal calculi, the largest measuring 3.7 mm, and bilateral medullary NC without hydroureter or debris in the bladder.

Twin B was reported to have frequent unexplained inconsolable crying spells. He was initially treated with Polycitra K and pyridoxine following the discovery of NC and was later started on lumasiran at 12 months of age following the gene confirmation of Twin A. His crying episodes stopped after starting lumasiran, and serum creatinine decreased to 0.12 mg/dl. Repeat sonograms eight months after the start of lumasiran demonstrated stable findings without new stone formation. Both children tolerated lumasiran well, without any evidence of disease recurrence at eight months following the start of therapy.

The biochemical pathway of oxalate production in liver cells and lumasiran's mechanism of action are illustrated in Figure 1.

## Discussion

We described a case of twin males with infantile PH1 treated with an RNAi, lumasiran. To the best of our knowledge, PH1 has not been reported in twins before. Moreover, these may be the youngest children with PH1 who are reported to have received lumasiran following the FDA approval. Most of the available literature on lumasiran involves children aged >6 years. PH1 in twins serves as an illustrative case of the phenotypic variability of this disease. Despite having identical mutations, the clinical severity of the disease and clinical manifestations were different. Following the start of lumasiran, both children had almost immediate improvement in their overall wellbeing.

PH1 is a rare genetic disease associated with kidney stones and approximately 1% of pediatric ESKD registries in the United States, Europe, and Japan [2]. In PH1, stones have a composition of >95% calcium oxalate monohydrate (whewellite) [1]. In our patients, the composition was only 80%. Both patients demonstrated higher-than-normal oxalate levels on random urine oxalate-creatinine ratio. Oxalosis affecting the skin was described as painful skin nodules, skin necrosis, gangrene, calciphylaxis-like skin lesions, and pruritus [2]; however, Twin A was noted to have repeated hives triggered by activities and certain situations. These became less frequent after the start of lumasiran.

Prior to the development of RNAi, treatment options for PH1 were limited, and the management focused on the goal of decreasing kidney stone formation and slowing disease progression. The main treatment options included hyperhydration, high-dose pyridoxine, and calcium oxalate crystallization inhibitors [4]. Our patients were started on pyridoxine and Polycitra K, which were discontinued after the start of lumasiran.

The twin boys demonstrated significant improvement in their kidney function despite the early presentation with a high stone burden. There was no metabolic acidosis or failure to thrive in either of them when weights were adjusted to their gestational ages. This may be related to their genetic mutation consisting of two pathogenic variants of AGXT, which can present milder cases [8]. Fatal infantile cases have been reported in the literature with infants presenting in their early months with chronic kidney disease (CKD) and ESKD by the first year of life. All of these patients have been reported to do poorly on dialysis and subsequently died [9,10]. A combined liver-kidney transplant is recommended for those with organ failure as dialysis can be challenging in these patients [1,2].

Another RNAi is being investigated for the potential treatment of all three forms of PH by blocking hepatic lactate dehydrogenase A. Phase I clinical data are available on nedosiran as a safe alternative that will be further investigated [11].

## Conclusions

To the best of our knowledge, this is the first report on infantile PH1 in twins. Both infants had bilateral NC, although one of them was more affected than the other. They may also be the youngest children reported to be treated with lumasiran so far since its FDA approval. They had a dramatic clinical response to the therapy with increased overall wellbeing and quality of life.
